# Effects of green tea consumption on cognitive dysfunction in an elderly population: a randomized placebo-controlled study

**DOI:** 10.1186/s12937-016-0168-7

**Published:** 2016-05-04

**Authors:** Kazuki Ide, Hiroshi Yamada, Norikata Takuma, Yohei Kawasaki, Shohei Harada, Junpei Nakase, Yuuichi Ukawa, Yuko M. Sagesaka

**Affiliations:** 1 Department of Drug Evaluation & Informatics, Graduate school of Pharmaceutical Sciences, University of Shizuoka, 52-1 Yada, Suruga-ku, Shizuoka, 422-8526 Japan; 2 White Cross Nursing Home, 2-26-1 Suwa-cho, Higashimurayama, Tokyo 189-0021 Japan; 3 Central Research Institute, ITO EN, Ltd., 21 Mekami, Makinohara, Shizuoka 421-0516 Japan

**Keywords:** Green tea, Oral administration, Cognitive function, Elderly, Randomized-controlled trial

## Abstract

**Background:**

Green tea is a beverage with potential effects on cognitive dysfunction, as indicated by results of experimental studies. However, its effects in humans, especially at real-world (typical) consumption levels, are unclear.

**Methods:**

A double-blind, randomized controlled study was conducted to assess the effects of green tea consumption on cognitive dysfunction (Mini-Mental State Examination Japanese version (MMSE-J) score <28) in Japan. Participants were randomly allocated to the green tea or placebo group, and consumed either 2 g/day of green tea powder (containing 220.2 mg of catechins) or placebo powder (containing 0.0 mg of catechins), respectively, for 12 months. Cognitive function assessments were performed every 3 months using the MMSE-J and laboratory tests.

**Results:**

Thirty-three nursing home residents with cognitive dysfunction were enrolled (four men, 29 women; mean age ± SD, 84.8 ± 9.3; mean MMSE-J score ± SD, 15.8 ± 5.4), of whom 27 completed the study. Changes of MMSE-J score after 1 year of green tea consumption were not significantly different compared with that of the placebo group (−0.61 [−2.97, 1.74], least square mean (LSM) difference [95 % CI]; *P* = 0.59). However, levels of malondialdehyde-modified low-density lipoprotein (U/L), a marker of oxidative stress, was significantly lower in the green tea group (−22.93 [−44.13, −1.73], LSM difference [95 % CI]; *P* = 0.04).

**Conclusions:**

Our results suggest that 12 months green tea consumption may not significantly affect cognitive function assessed by MMSE-J, but prevent an increase of oxidative stress in the elderly population. Additional long-term controlled studies are needed to clarify the effects.

**Trial registration:**

UMIN000011668

**Electronic supplementary material:**

The online version of this article (doi:10.1186/s12937-016-0168-7) contains supplementary material, which is available to authorized users.

## Background

With the increasing age of the global population, the prevalence of age-related diseases, especially dementia is increasing [1].

Globally, dementia affects 5 to 7 % of individuals over 60 years old [2]. Alzheimer’s disease International reported 44.35 million people with dementia in 2013 worldwide, and estimated that this number nearly doubles every 20 years. Their updated report estimated a total of 135.46 million people with dementia by 2050 [3]. Despite the availability of medications, such as cholinesterase inhibitors and memantine, no curative therapy is currently available [1, 4, 5]. Additionally, no candidate drug for dementia treatment has been approved since memantine’s approval in 2002 for Europe and 2003 for the U.S. [5]. Given this situation, preventing dementia and limiting its progression with a combination of pharmaceutical and non-pharmaceutical treatments are important to improve the quality of life of the elderly. A number of epidemiological studies suggest that lifestyle, including dietary and nutritional factors, is related to risk of dementia [6–9]. In addition to these studies and the pathophysiology of dementia suggests that the modification of oxidative stress is important for preventing the disease and limiting its progression [8, 10, 11].

Green tea is a beverage with anti-oxidative stress and neuromodulating properties, as revealed by experimental studies in vitro and in vivo [12–14]; this is also supported by clinical studies [15, 16]. Epidemiological studies, including cross-sectional and longitudinal studies, focusing on cognitive function, have yielded contrasting results. Some found a relationship between green tea consumption and the prevalence of dementia and/or cognitive decline [17–21], while others did not find a correlation at all, or identified sex-related differences [22–25]. In addition, only 3 intervention studies on the effect of green tea consumption on cognitive dysfunction were conducted [26–28]. All of these intervention studies suggested that green tea or green tea-based dietary supplements may reduce the rate of cognitive decline or even improve cognitive function. However, one of them used green tea-based supplements [28], and the results of other two studies were inconclusive [26, 27].

In a previous pilot intervention study [26], we used normal green tea powder. Participants consumed 2 g/day of the powder (approximately equal to two to four cups of tea/day). Improvement of cognitive function, as evaluated by the Mini-Mental State Examination Japanese version (MMSE-J), was measured after 3 months of daily consumption. The results suggest potential effects of typical green tea consumption on cognitive dysfunction. However, the design of the pilot study was a before-after trial, and 3 months of study period was too short to reliably measure the effects. Additional studies with a longer study period are thus needed.

Based on this, we conducted a randomized, placebo-controlled study to evaluate the effects of 12 months of green tea consumption on cognitive dysfunction and related risk factors in the elderly.

## Methods

### Design overview

A randomized placebo-controlled study was conducted to evaluate the effect of green tea consumption on cognitive dysfunction in the elderly. The study took place at White Cross Nursing Home in Higashi-murayama, Tokyo, Japan. Thirty-three participants were enrolled to consume 2 g/day of green tea or placebo powder for 12 months. The MMSE-J, for assessments of cognitive function, and laboratory tests, for related risk factors, were administered before the intervention, and then every 3 months. Changes in MMSE-J scores and laboratory test results were compared between the green tea and the placebo groups. The supporting CONSORT checklist are available as supporting information; see Additional file 1.

### Setting and participants

Participant recruitment was performed at the nursing home by posters and announcements. The inclusion criteria were as follows: 1) ≥50 years of age; 2) ability to orally ingest green tea and placebo powder; 3) No consumption of food supplements with anti-oxidative stress effects (vitamins E, C, A, and β-carotene) during the study period; and 4) An MMSE-J score of <28 [29]. The exclusion criteria were as follows: 1) tea allergy; 2) severe cardiac, respiratory, hepatic, or renal dysfunction; and 3) severe anemia. The MMSE-J was administered to all applicants to evaluate the inclusion criteria and assess the baseline cognitive performance.

The following baseline clinical characteristics were recorded by the nursing home medical staff for each participant: age, sex, body mass index (BMI), underlying disease, complications, medications, alcohol consumption, smoking habits, tea and supplement consumption habits, activity of daily living, and brain magnetic resonance imaging (MRI) or computed tomography (CT). A tea consumption habit was defined as ≥1 cup/day.

### Randomization and interventions

Eligible participants were randomized by a computer generated permuted block randomized schema, and stratified according to the nursing home building. A 1:1 allocation ratio was used. The randomization process was performed at the Data Management Center of Shizuoka General Hospital in Japan.

The participants orally consumed 2.0 g/day of green tea or placebo powder. The green tea powder contained 220.2 mg of catechins (gallocatechin, 10.9 mg; epigallocatechin, 72.0 mg; catechin, 2.0 mg; epicatechin, 22.0 mg; epigallocatechin gallate, 88.0 mg; gallocatechin gallate, 8.0 mg; epicatechin gallate, 17.3 mg), 20.8 mg of theanine, and 60.0 mg of caffeine. The placebo powder consisted of 1.6 g of cellulose powder and 0.4 g of gardenia/caramel pigment and contained 0.0 mg of catechins. Both powders were manufactured by ITO EN Ltd. (Tokyo, Japan). The nursing home staff maintained a daily compliance report.

All participants and/or their legal representatives gave written informed consent before entering the study.

### Outcome and follow-up

The primary outcome measure was the difference of MMSE-J score changes from the baseline after 12 months of intervention between the green tea and the placebo groups.

Several secondary outcome measures were also examined. Score changes from the baseline were compared using last observation carried forward (LOCF) data. Changes of MMSE-J scores measured every 3 months were also compared between groups. The Neuropsychiatric Inventory Questionnaire (NPI-Q) was administered to assess behavioral and psychological symptoms of dementia (BPSD) [30] and was rated by caregivers (care-giving staff in the nursing home). The following data were also collected: blood pressure; serum lipid levels (Total cholesterol [TC], low-density lipoprotein cholesterol [LDL-C], high-density lipoprotein cholesterol [HDL-C], triglycerides [TG], and malondialdehyde-modified low-density lipoprotein [MDA-LDL]); blood glucose levels (fasting plasma glucose [FPG]); and hemoglobin A1c (HbA1c). All outcome measures were assessed before intervention (baseline) and every three months during the study period.

### Statistical analyses

Based on our previous study and studies using the MMSE [26, 31–33], we estimated the mean difference of MMSE-J scores between the two groups 12 months after starting the intervention as 1.7 points, with 1.4 points of standard deviation (SD). Sample size was estimated as 12 for each group, with a power level of 0.80 to detect a difference of MMSE score changes and a 2-sided alpha level of 0.05. Given the nature of the tested population (elderly people with cognitive dysfunction), dropout rate was estimated as 25 %. We thus set our total sample size at 32.

Both the full analysis set (FAS) and the per-protocol set (PPS) were used for all efficacy and safety analyses. No interim analysis was planned. The FAS was determined by excluding participants from the intention-to-treat population according to the following criteria: 1) no green tea or placebo consumption, and/or 2) no intervention data collected, or withdrawal from the study with or without refusal to have data included in the study. In addition to these criteria, the PPS was defined with the following criteria: 1) adherence rate of green tea or placebo consumption at or above 90 %.

Continuous variables were expressed as mean ± SD, while categorical variables were expressed as numbers and percentages of the group. Differences in the mean values of continuous measurements were tested by analysis of covariance (ANCOVA) [34] with age as a covariate because of its medical implication [10]. A repeated measures ANCOVA was also performed to analyze changes of MMSE-J scores from the baseline up to 12 months after starting the intervention [35]. In this analysis, age was also considered a covariate. The results of the ANCOVA and repeated measures ANCOVA were expressed as least square mean (LSM) ± standard error (SE), while the difference between the two groups was expressed as LSM difference with 95 % confidence interval (95 % CI) [36]. LOCF was also performed for the analysis of MMSE-J scores.

Statistical significance was set to *P* < 0.05, and all statistical analyses were performed using SAS version 9.4 for Windows (SAS Institute Inc., Cary, NC, USA).

### Ethics statement

The study protocol was approved by the ethics committee of the University of Shizuoka (No. 25–10; approved on June 25, 2013) and was conducted in accordance with the Declaration of Helsinki. The study was registered at the University hospital Medical Information Network (UMIN) as #UMIN000011668.

## Results

### Study population

**Fig. 1:**
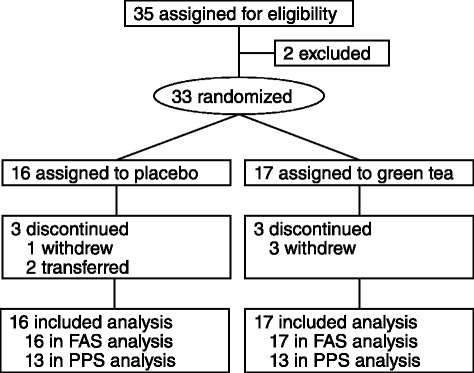
Study flow diagram

The flow diagram for this study is shown in Fig. 1. Thirty-five nursing home residents or their legal representatives gave written informed consent. Upon assessment of eligibility, two residents were excluded based on the exclusion criteria. Thirty-three nursing home residents with cognitive dysfunction were enrolled (4 men, 29 women; mean age ± SD, 84.8 ± 9.3 years), and allocated randomly to a group: 17 participants to the green tea group, and 16 to the placebo group. Twenty-seven participants completed the study, as 6 participants discontinued: 4 withdrew by themselves (1 from the placebo group and 3 from the green tea group) and 2 were transferred to a hospital (both from the placebo group). According to the criteria described in the Methods section, 33 participants were included in the FAS, and 26 in the PPS. In the PPS, 1 participant was excluded because of his/her low adherence rate (<90 %) in addition to the 6 participants who discontinued.

### Baseline characteristics

Participants’ baseline characteristics are shown in Table 1. The mean age of participants was 84.8 ± 9.3 years (placebo group, 87.9 ± 5.6 years; green tea group, 81.8 ± 11.1 years). Seventeen participants had Alzheimer’s disease (8 in the placebo group, 9 in the green tea group), 15 had vascular dementia (8 in the placebo group, 7 in the green tea group), and 1 in the green tea group had dementia with Lewy bodies. Among 33 participants, 5 were being treated with medications for dementia (3 in the placebo group, and 2 in the green tea group), and the doses of medications were not changed during the intervention period. All 33 participants habitually drank tea (≥1 cup/day), and none of the participants had a habit of consuming any type of dietary supplements. Total adherence rate for green tea and placebo consumption was 89.6 ± 23.4 % in the FAS (placebo group, 90.7 ± 22.4 %; green tea group, 88.7 ± 25.0 %), and 99.0 ± 1.9 % in the PPS (placebo group, 99.5 ± 0.8 %; green tea group, 98.5 ± 2.6 %).

**Table 1:** Clinical characteristics of participants

	Placebo	Green tea
Number of subjects	16	17
Age, mean ± SD	87.9 ± 5.6	81.8 ± 11.1
Sex, n (%)		
Men	1 (6.3)	3 (17.6)
Women	15 (93.7)	14 (82.4)
BMI, mean ± SD	21.6 ± 3.3	22.6 ± 2.8
Underlying diseases, n (%)		
Alzheimer’s disease	8 (50.0)	9 (52.9)
Vascular dementia	8 (50.0)	7 (41.2)
Dementia with Lewy bodies	0 (0.0)	1 (5.9)
CT/MRI		
CT	15 (93.8)	17 (100.0)
CT, MRI	1 (6.2)	0 (0.0)
MMSE-J score, mean ± SD	15.7 ± 4.4	15.9 ± 6.3
Complications, n (%)		
Hypertension	9 (56.3)	13 (76.5)
Dyslipidaemia	7 (43.8)	3 (17.6)
Diabetes	4 (25.0)	5 (29.4)
Concomitant drugs, n (%)		
Antihypertensive drugs	12 (75.0)	13 (76.5)
Antidyslipidemic drugs	5 (31.3)	2 (11.8)
Antidiabetic drugs	4 (25.0)	4 (23.5)
Dementia therapeutic drugs	3 (18.8)	2 (11.8)
Activities of daily living, n (%)		
Independent	4 (25.0)	5 (29.4)
Some assistance necessary	12 (75.0)	11 (64.7)
Full assistance necessary	0 (0.0)	1 (5.9)
Daily tea consumption	16 (100.0)	17 (100.0)
Green tea, n (%)	16 (100.0)	17 (100.0)
Other, n (%)	5 (31.3)	7 (41.2)
Alcohol use, n (%)	0 (0.0)	1 (5.9)
Smoking, n (%)	1 (6.3)	2 (11.8)
Dietary supplement consumption, n (%)	0 (0.0)	0 (0.0)

*BMI* body mass index, *CT* computed tomography, *MRI* Magnetic resonance imaging, *MMSE-J* Mini Mental State Examination Japanese version, *SD* standard deviation

### Mini-mental state examination

Changes in MMSE-J scores during the study period are shown in Table 2. The score difference after 1 year of green tea consumption were not significantly different compared with the placebo group (FAS: −0.61 [−2.97, 1.74], LSM difference [95 % CI]; *P* = 0.59. PPS: −0.58 [−3.08, 1.92], *P* = 0.64) (Table 3). The results of the analysis with LOCF (FAS: −0.59 [−2.62, 1.45], LSM difference [95 % CI]; *P* = 0.56. PPS: −0.67 [−3.10, 1.76], *P* = 0.57) and the repeated measures ANCOVA (FAS: −0.33 ± 0.94, LSM difference ± SE; *P* = 0.73. PPS: −0.67 ± 1.00, *P* = 0.51) did not show a significant difference either (Table 3).

**Table 2:** MMSE-J score changes

FAS	Baseline	3 months	6 months	9 months	12 months	LOCF
Total MMSE-J, mean ± SD						
Placebo	15.7 ± 4.4	16.1 ± 5.5	16.8 ± 5.2	18.8 ± 4.8	17.7 ± 5.2	16.4 ± 5.3
Green tea	15.9 ± 6.3	15.9 ± 6.4	17.4 ± 6.3	16.0 ± 6.5	16.5 ± 7.2	16.1 ± 6.4
PPS						
Total MMSE-J, mean ± SD	Baseline	3 months	6 months	9 months	12 months	LOCF
Placebo	16.7 ± 4.2	17.1 ± 5.2	17.5 ± 4.6	19.7 ± 3.9	17.7 ± 5.2	17.7 ± 5.2
Green tea	15.2 ± 6.4	15.2 ± 6.3	17.2 ± 6.4	15.2 ± 6.3	15.7 ± 7.0	15.5 ± 6.7

*FAS* full analysis set, *SD* standard deviation, *LOCF* last observation carried forward, *MMSE-J* Mini Mental State Examination, Japanese version, *PPS* per protocol set

**Table 3:** ANCOVA and repeated measures ANCOVA analysis

	12 months			LOCF		
ANCOVA	Placebo	Green tea	*P*-value	Placebo	Green tea	*P*-value
FAS	*N* = 16	*N* = 17		*N* = 16	*N* = 17	
Total MMSE-J Score, LSM ± SE	1.04 ± 0.80	0.42 ± 0.77	0.59	0.71 ± 0.71	0.13 ± 0.66	0.56
LSM difference [95 % CI]	−0.61 [−2.97, 1.74]			−0.59 [−2.62, 1.45]		
PPS	*N* = 13	*N* = 13		*N* = 13	*N* = 13	
Total MMSE-J Score, LSM ± SE	1.04 ± 0.83	0.46 ± 0.83	0.64	1.03 ± 0.82	0.36 ± 0.79	0.57
LSM mean difference [95 % CI]	−0.58 [−3.08, 1.92]			−0.67 [−3.10, 1.76]		
Repeated measures ANCOVA						
FAS	LSM ± SE	LSM difference ± SE	*P*-value			
Placebo	0.75 ± 0.68	−0.33 ± 0.94	0.73			
Green tea	0.42 ± 0.63					
PPS						
Placebo	1.16 ± 0.70	−0.67 ± 1.00	0.51			
Green tea	0.49 ± 0.67					

*ANCOVA* analysis of covariance, *CI* confidence interval, *FAS* full analysis set, *LOCF* last observation carried forward, *LSM* least square mean, *MMSE-J* Mini Mental State Examination, Japanese version, *PPS* per protocol set, *SD* standard deviation, *SE* standard error

### Neuropsychiatric inventory questionnaire and laboratory test

NPI-Q scores and laboratory test results are shown in Tables 4 and 5, Additional file 2: Tables S1 and Additional file 3: Table S2. The baseline symptoms and distress scores of the NPI-Q were low in both groups and scores after 12 months of intervention were not significantly different between the two groups (FAS: Symptom, −0.34 [−2.52, 1.85], LSM difference [95 % CI]; distress, −0.25 [−2.17, 1.67]; *P* = 0.79. PPS: Symptom, −0.46 [−2.84, 1.92]; distress, −0.43 [−2.49, 1.64]). Among laboratory test values, MDA-LDL level, which is a marker of oxidative stress, was significantly maintained lower in the green tea group (FAS: −22.93 [−44.13, −1.73], LSM difference [95 % CI]; *P* = 0.04. PPS: −24.50 [−46.84, −2.16]; *P* = 0.03). Blood pressure (FAS, *P* = 0.76 for systolic blood pressure (SBP), *P* = 0.74 for diastolic blood pressure (DBP); PPS, *P* = 0.72 for SBP, *P* = 0.71 for DBP), other serum lipid status TC (FAS, *P* = 0.20; PPS, *P* = 0.38), HDL-C (FAS, *P* = 0.96; PPS, *P* = 0.89), LDL-C (FAS, *P* = 0.14; PPS, *P* = 0.28) and TG (FAS, *P* = 0.65; PPS, *P* = 0.26), as well as blood glucose levels (FAS, *P* = 0.81 for FPG, *P* = 0.16 for HbA1c; PPS, *P* = 0.99 for FPG, *P* = 0.27 for HbA1c) were not significantly different between the green tea and placebo groups.

**Table 4:** Values of laboratory tests during study period

FAS	Baseline	3 months	6 months	9 months	12 months
NPI-Q: Total symptom score, mean ± SD					
Placebo	1.7 ± 2.3	2.0 ± 2.8	2.4 ± 3.0	1.9 ± 2.0	2.6 ± 3.2
Green tea	3.1 ± 4.4	2.7 ± 3.1	2.7 ± 3.6	2.0 ± 2.4	1.6 ± 2.0
NPI-Q: Total distress score, mean ± SD					
Placebo	1.7 ± 2.3	1.3 ± 2.5	2.2 ± 3.5	2.0 ± 3.7	2.1 ± 3.0
Green tea	3.2 ± 4.5	1.9 ± 2.6	2.5 ± 4.5	2.1 ± 3.5	1.3 ± 1.6
Blood pressure					
SBP (mmHg), mean ± SD					
Placebo	122.5 ± 17.8	125.3 ± 14.1	120.5 ± 17.3	116.2 ± 14.6	122.0 ± 16.3
Green tea	125.6 ± 15.7	125.4 ± 17.0	129.1 ± 23.7	125.0 ± 13.5	121.3 ± 12.2
DBP (mmHg), mean ± SD					
Placebo	69.3 ± 10.3	72.3 ± 11.7	65.9 ± 11.3	65.1 ± 13.6	66.8 ± 10.9
Green tea	73.4 ± 10.7	72.8 ± 12.6	75.5 ± 14.0	68.3 ± 10.4	70.7 ± 8.0
Serum lipid levels					
TC (mg/dL), mean ± SD					
Placebo	185.7 ± 20.3	188.7 ± 20.7	189.6 ± 24.3	187.9 ± 25.1	184.7 ± 27.8
Green tea	180.4 ± 36.3	182.1 ± 35.3	178.8 ± 32.6	176.0 ± 34.9	169.4 ± 25.9
HDL-C (mg/dL), mean ± SD					
Placebo	46.4 ± 9.2	47.7 ± 8.0	50.6 ± 9.7	48.1 ± 8.4	47.0 ± 8.9
Green tea	47.4 ± 11.0	48.6 ± 11.3	50.1 ± 11.0	50.9 ± 10.5	47.8 ± 9.5
LDL-C (mg/dL), mean ± SD					
Placebo	114.8 ± 19.5	114.1 ± 18.8	113.6 ± 21.3	114.4 ± 21.5	111.1 ± 24.0
Green tea	110.2 ± 30.4	107.4 ± 28.8	105.4 ± 29.5	100.9 ± 29.4	95.0 ± 22.1
TG (mg/dL), mean ± SD					
Placebo	127.8 ± 52.7	115.4 ± 34.9	106.3 ± 34.2	103.7 ± 39.4	115.7 ± 42.6
Green tea	122.6 ± 48.9	117.0 ± 45.1	106.7 ± 31.7	92.7 ± 37.5	117.1 ± 53.2
MDA-LDL (U/L), mean ± SD					
Placebo	84.3 ± 14.7	86.8 ± 16.6	100.1 ± 25.2	90.0 ± 18.1	100.9 ± 20.0
Green tea	88.3 ± 35.1	89.9 ± 29.8	94.5 ± 26.7	88.9 ± 37.5	89.9 ± 19.9
Blood glucose levels					
FPG (mg/dL), mean ± SD					
Placebo	115.4 ± 37.3	118.1 ± 35.3	105.4 ± 26.9	115.7 ± 33.7	112.0 ± 32.1
Green tea	116.8 ± 34.0	110.8 ± 30.8	120.0 ± 38.5	119.9 ± 26.7	126.9 ± 41.2
HbA1c (%), mean ± SD					
Placebo	5.6 ± 0.6	5.6 ± 0.7	5.6 ± 0.6	5.6 ± 0.5	5.6 ± 0.5
Green tea	5.5 ± 0.4	5.6 ± 0.5	5.6 ± 0.5	5.6 ± 0.5	5.6 ± 0.6

*DBP* diastolic blood pressure, *FAS* full analysis set, *FPG* fasting plasma glucose, *HbA1c* hemoglobin A1c, *HDL-C* high-density lipoprotein cholesterol, *LDL-C* low-density lipoprotein cholesterol, *LSM* least square mean, *MDM-LDL* malondialdehyde-modified low-density lipoprotein, *NPI-Q* Neuropsychiatric Inventory Questionnaire, *SBP* systolic blood pressure, *SD* standard deviation, *TC* total cholesterol, *TG* triglycerides

**Table 5:** ANCOVA and repeated measures ANCOVA analysis for secondary outcomes

	Placebo	Green tea	*P*-value
FAS analysis	*N* = 16	*N* = 17	
Total MMSE Score, LSM ± SE	1.04 ± 0.80	0.42 ± 0.77	0.59
LSM difference [95 % CI]	−0.61 [−2.97, 1.74]		
NPI-Q: Total symptom score, LSM ± SE	0.56 ± 0.74	0.23 ± 0.74	0.76
LSM difference [95 % CI]	−0.34 [−2.52, 1.85]		
NPI-Q: Total distress score, LSM ± SE	−0.02 ± 0.64	−0.27 ± 0.64	0.79
LSM difference [95 % CI]	−0.25 [−2.17, 1.67]		
Blood pressure			
SBP (mmHg), LSM ± SE	−1.94 ± 4.88	−4.06 ± 4.69	0.76
LSM difference [95 % CI]	−2.12 [−16.46, 12.22]		
DBP (mmHg), LSM ± SE	−1.15 ± 3.71	−2.93 ± 3.57	0.74
LSM difference [95 % CI]	−1.79 [−12.69, 9.12]		
Serum lipid levels			
TC (mg/dL), LSM ± SE	0.41 ± 5.72	−10.23 ± 5.50	0.20
LSM difference [95 % CI]	−10.64 [−27.46, 6.18]		
HDL-C (mg/dL), LSM ± SE	0.69 ± 1.75	0.57 ± 1.68	0.96
LSM difference [95 % CI]	−0.12 [−5.26, 5.02]		
LDL-C (mg/dL), LSM ± SE	−4.26 ± 4.92	−14.90 ± 4.73	0.14
LSM difference [95 % CI]	−10.63 [−25.09, 3.83]		
TG (mg/dL), LSM ± SE	−8.80 ± 11.59	−1.32 ± 11.15	0.65
LSM difference [95 % CI]	7.48 [−26.60, 41.57]		
MDA-LDL (U/L), LSM ± SE	21.52 ± 7.21	−1.41 ± 6.93	0.04
LSM difference [95 % CI]	−22.93 [−44.13, −1.73]		
Blood glucose levels			
FPG (mg/dL), LSM ± SE	0.75 ± 10.46	4.38 ± 10.06	0.81
LSM difference [95 % CI]	3.63 [−27.13, 34.38]		
HbA1c (%), LSM ± SE	0.16 ± 0.08	0.01 ± 0.07	0.16
LSM difference [95 % CI]	−0.16 [−0.38, 0.07]		

*ANCOVA* analysis of covariance, *CI* confidence interval, *DBP* diastolic blood pressure, *FAS* full analysis set, *FPG* fasting plasma glucose, *HbA1c* hemoglobin A1c, *HDL-C* high-density lipoprotein cholesterol, *LDL-C* low-density lipoprotein cholesterol, *LSM* least square mean, *MDM-LDL* malondialdehyde-modified low-density lipoprotein, *NPI-Q* Neuropsychiatric Inventory Questionnaire, *SBP* systolic blood pressure, *SE* standard error, *TC* total cholesterol, *TG* triglycerides

No serious adverse events associated with the intervention were observed during the study period.

## Discussion

This randomized study was conducted to evaluate the effects of 12-month green tea consumption on cognitive dysfunction and related risk factors in the elderly. Twelve-month green tea consumption significantly maintained lower levels of oxidative stress marker MDA-LDL, but the MMSE-J score was not improved significantly.

MMSE-J scores were stable in each group during the study period, and this is a main limitation of this study. Green tea is not a medication, and our study used a dose that can be taken in daily-life situations. A 2 g/day intake of green tea powder (containing 220.2 mg of catechins) is equivalent to 2 to 4 cups/day of bottled or home-brewed green tea consumption [37–39]. Therefore, to know the effects of green tea consumption on cognitive dysfunction, long-term interventions that can follow cognitive decline in the placebo population may be needed. Our previous study used a before-after trial design [26], and a randomized study by other researchers used green tea-based supplements [28]. This may have led to the discrepant results. In addition, all of the participants of this study were regular tea drinker, and tea consumption during the study period was not restricted because of ethical reasons. Therefore, baseline tea consumption might affect the changes and the differences of MMSE-J scores in both groups. In this study, the MMSE-J was administered every 3 months. Tombaugh [40] reported test-retest reliability of the MMSE with 3-month test intervals. In this study, the MMSE was administered to individuals aged 65 to 89 at 3-month intervals; the mean score changes for the first and second 3-month periods were 0.69 and 0.35 points, respectively. Thus, changes in MMSE scores caused by practice effects are unlikely to affect results over 3-month intervals, and therefore the test interval used in this study is appropriate for the identification of cognitive changes related to the intervention.

Unexpectedly, our study did not reveal effects of green tea consumption on cognitive function, in spite of our attempt to improve upon our previous before-after study design by performing a randomized placebo-controlled study. Our results showed that MMSE-J scores were not significantly different between the groups. In addition to the non-restricted baseline tea consumption during the study period, the sex of the study subjects and the different types of dementia present in these patients are factors that may have interfered with the results, despite the even distribution of sexes and dementia patients between the experimental groups.

However, MDA-LDL, which is an oxidative stress marker in peripheral blood, was significantly maintained lower in the green tea group. Oxidative stress is considered to be involved in late-onset neurodegenerative disorders [41–43]. It is caused by reactive oxygen species, which induce cellular dysfunction and degeneration interacting with biomolecules [44]. Cellular dysfunction and degeneration in the brain leads to progressive neurodegenerative disorders including dementia. Additionally, an increase of antibodies against MDA-LDL has been reported in the cerebrospinal fluid in people with Alzheimer’s disease, and may be related to the pathophysiology of dementia [45]. Antioxidative stress properties of green tea and its ingredients have been reported in experimental and clinical studies [13, 15, 46].

A study involving men of age range 24–46 found MDA-LDL reduction by green tea consumption [46], but to our knowledge, our study is the first one to report this effect in the elderly. It is possible that tea catechins are involved in the process and that 12 months of green tea consumption can indeed prevent an increase in MDA-LDL in elderly individuals with cognitive dysfunction. This result suggests that green tea has the potential to prevent or treat dementia through combination therapy with pharmaceutical and/or non-pharmaceutical interventions. Neither the medical histories of the participants nor their levels of medical care changed during the study period. Therefore, the significant reduction in oxidative stress status in the green tea group was likely caused solely by green tea consumption. This difference was observed between groups of participants who habitually drank tea; therefore, this effect may also be observed in regular green tea drinkers. All of the participants of this study consumed at least one cup of green tea, equivalent to approximately 50 to 100 mg of catechins [37–39], per day. Although this baseline level of catechin ingestion may not significantly affect oxidative stress status, it may be related to the minimal changes in MMSE-J scores observed in both groups.

NPI-Q and other laboratory tests were also performed in the study, but we did not find any significant difference between groups. In terms of NPI-Q scores, almost all of participants did not have BPSD or had only mild symptoms, and NPI-Q scores were low when starting intervention. Blood pressure, serum lipid levels, and blood glucose levels were also assessed. These clinical characteristics were not considered in the inclusion criteria but were measured from the starting point of the study. In future investigations, we will attempt to mitigate these confounding factors as much as possible. To this end, additional inclusion criteria, such as participants with dyslipidemia or other diseases, may be necessary in order to reveal the effects of green tea consumption on cognitive dysfunction and related risk factors. Considering these facts, long-term intervention and observation over a year may be needed to clarify the effects of normal daily green tea consumption on cognitive dysfunction in the elderly.

## Conclusions

Our results suggest that 12-month green tea consumption may not significantly affect cognitive function assessed by the MMSE-J. However, it may prevent oxidative stress increase in the elderly. Additional long-term controlled studies are needed to clarify the nature of this effect.

## Additional files

Additional file 1: CONSORT 2010 checklist of information to include when reporting a randomised trial. (DOC 217 kb)

Additional file 2: Table S1. Values of laboratory tests during study period in PPS. (DOCX 14 kb)

Additional file 3: Table S2. ANCOVA and repeated measures ANCOVA analysis in PPS. (DOCX 14 kb)

## Abbreviations

ANCOVA: analysis of covariance
BPSD: behavioral and psychological symptoms of dementia
DBP: diastolic blood pressure
FAS: full analysis set
FPG: fasting plasma glucose
HbA1C: hemoglobin A1c
HDL-C: high-density lipoprotein cholesterol
LDL-C: low-density lipoprotein cholesterol
LOCF: last observation carried forward
LSM: least square mean
MDA-LDL: malondialdehyde-modified low-density lipoprotein
MMSE-J: Mini-Mental State Examination Japanese version
NPI-Q: Neuropsychiatric Inventory Questionnaire
PPS: per-protocol set
SBP: systolic blood pressure
TC: total cholesterol
TG: triglycerides
