# Supplementary material for: Effects of green tea consumption on cognitive dysfunction in an elderly population: a randomized placebo-controlled study
Source: Nutr J. 2016 May 4;15:49. doi: 10.1186/s12937-016-0168-7 (PMC4855797; doi:10.1186/s12937-016-0168-7)
Supplement: Supplementary file 2 — Values of laboratory tests during study period in PPS. (DOCX 14 kb) [file 12937_2016_168_MOESM2_ESM.docx]

**Table S1**. Values of laboratory tests during study period in PPS

| ***PPS*** | Baseline | | | 3 months | | | 6 months | | | 9 months | | | 12 months | | |
| --- | --- | --- | --- | --- | --- | --- | --- | --- | --- | --- | --- | --- | --- | --- | --- |
| NPI-Q: Total symptom score, mean ± SD | | | | | | | | | | | | | | | |
| Placebo | | 2.1 ± 2.4 | | | 2.4 ± 3.0 | | | 2.3 ± 3.1 | | | 1.9 ± 2.1 | | | 2.6 ± 3.3 | |
| Green tea | | 1.6 ± 2.0 | | | 2.0 ± 2.2 | | | 2.2 ± 2.6 | | | 1.5 ± 1.9 | | | 1.5 ± 2.0 | |
| NPI-Q: Total distress score, mean ± SD | | | | | | | | | | | | | | | |
| Placebo | | 2.0 ± 2.4 | | | 1.5 ± 2.8 | | | 2.4 ± 3.5 | | | 2.1 ± 3.8 | | | 2.2 ± 3.1 | |
| Green tea | | 1.8 ± 2.6 | | | 1.8 ± 2.4 | | | 1.8 ± 3.0 | | | 1.6 ± 2.9 | | | 1.2 ± 1.6 | |
| Blood pressure | | | | | | | | | | | | | | | |
| SBP (mmHg), mean ± SD | | | | | | | | | | | | | | | |
| Placebo | | | 121.8 ± 16.3 | | | 124.0 ± 15.0 | | | 120.0 ± 17.9 | | | 117.3 ± 14.6 | | | 122.0 ± 16.3 |
| Green tea | | | 127.6 ± 17.4 | | | 122.7 ± 16.3 | | | 127.3 ± 26.1 | | | 122.1 ± 12.4 | | | 120.6 ± 12.4 |
| DBP (mmHg), mean ± SD | | | | | | | | | | | | | | | |
| Placebo | | | 67.1 ± 8.1 | | | 70.1 ± 12.0 | | | 66.3 ± 11.7 | | | 65.6 ± 14.0 | | | 66.8 ± 10.9 |
| Green tea | | | 75.1 ± 11.7 | | | 71.8 ± 13.0 | | | 78.0 ± 14.2 | | | 69.8± 10.2 | | | 70.9 ± 8.2 |
| Serum lipid levels | | | | | | | | | | | | | | | |
| TC (mg/dL), mean ± SD | | | | | | | | | | | | | | | |
| Placebo | | | 186.5 ± 22.5 | | | 191.8 ± 21.9 | | | 191.5 ± 24.2 | | | 189.2 ± 25.5 | | | 184.7 ± 27.8 |
| Green tea | | | 173.6 ± 36.1 | | | 174.5 ± 34.0 | | | 172.5 ± 32.9 | | | 172.9 ± 26.4 | | | 168.1 ± 26.4 |
| HDL-C (mg/dL), mean ± SD | | | | | | | | | | | | | | | |
| Placebo | | | 46.6 ± 8.2 | | | 48.4 ± 5.9 | | | 49.8 ± 9.7 | | | 47.6 ± 8.5 | | | 47.0 ± 8.9 |
| Green tea | | | 46.8 ± 11.0 | | | 47.0 ± 10.9 | | | 48.8 ± 10.5 | | | 49.5 ± 10.0 | | | 47.5 ± 9.9 |
| LDL-C (mg/dL), mean ± SD | | | | | | | | | | | | | | | |
| Placebo | | | 116.8 ± 20.8 | | | 117.0 ± 19.4 | | | 116.2 ± 19.9 | | | 116.2 ± 21.2 | | | 111.1 ± 24.0 |
| Green tea | | | 105.6 ± 31.0 | | | 102.5 ± 28.0 | | | 100.5 ± 29.2 | | | 99.8 ± 31.3 | | | 94.3 ± 22.8 |
| TG (mg/dL), mean ± SD | | | | | | | | | | | | | | | |
| Placebo | | | 125.5 ± 53.0 | | | 117.9 ± 34.6 | | | 108.2 ± 34.8 | | | 106.6 ± 39.4 | | | 115.7 ± 42.6 |
| Green tea | | | 110.8 ± 41.6 | | | 114.8 ± 47.7 | | | 111.7 ± 33.0 | | | 93.8 ± 39.2 | | | 117.5 ± 55.3 |
| MDA-LDL (U/L), mean ± SD | | | | | | | | | | | | | | | |
| Placebo | | | 81.2 ± 14.6 | | | 86.7 ± 17.9 | | | 103.2 ± 23.3 | | | 91.3 ± 18.2 | | | 100.9 ± 20.0 |
| Green tea | | | 90.2 ± 38.5 | | | 89.0 ± 30.8 | | | 91.1 ± 26.8 | | | 91.0 ± 34.0 | | | 90.0 ± 20.7 |
| Blood glucose levels | | | | | | | | | | | | | | | |
| FPG (mg/dL), mean ± SD | | | | | | | | | | | | | | | |
| Placebo | | | 113.7 ± 40.2 | | | 117.1 ± 34.1 | | | 106.8 ± 27.5 | | | 115.8 ± 35.0 | | | 112.0 ± 32.1 |
| Green tea | | | 121.3 ± 37.2 | | | 115.5 ± 32.5 | | | 123.8 ± 40.6 | | | 117.8 ± 27.9 | | | 126.2 ± 42.7 |
| HbA1c (%), mean ± SD | | | | | | | | | | | | | | | |
| Placebo | | | 5.5 ± 0.6 | | | 5.5 ± 0.7 | | | 5.6 ± 0.6 | | | 5.6 ± 0.5 | | | 5.6 ± 0.5 |
| Green tea | | | 5.5 ± 0.5 | | | 5.6 ± 0.5 | | | 5.6 ± 0.6 | | | 5.6 ± 0.6 | | | 5.6 ± 0.6 |

DBP, diastolic blood pressure; FAS, full analysis set; FPG, fasting plasma glucose; HbA1c, hemoglobin A1c;HDL-C, high-density lipoprotein cholesterol; LDL-C, low-density lipoprotein cholesterol; LSM, least square mean; MDM-LDL, malondialdehyde-modified low-density lipoprotein ; NPI-Q, Neuropsychiatric Inventory Questionnaire; SBP, systolic blood pressure; SD, standard deviation; TC, total cholesterol; TG, triglycerides
