# Supplementary material for: Effects of green tea consumption on cognitive dysfunction in an elderly population: a randomized placebo-controlled study
Source: Nutr J. 2016 May 4;15:49. doi: 10.1186/s12937-016-0168-7 (PMC4855797; doi:10.1186/s12937-016-0168-7)
Supplement: Supplementary file 3 — ANCOVA and repeated measures ANCOVA analysis in PPS. (DOCX 14 kb) [file 12937_2016_168_MOESM3_ESM.docx]

**Table S2**. ANCOVA and repeated measures ANCOVA analysis in PPS

|  | Placebo | Green tea | *P*-value |
| --- | --- | --- | --- |
| ***PPS analysis*** | *N* = 13 | *N* = 13 |  |
| Total MMSE Score, LSM ± SE | 1.04 ± 0.83 | 0.46 ± 0.83 | 0.64 |
| LSM difference [95% CI] | -0.58 [-3.08, 1.92] | |  |
| NPI-Q: Total symptom score, LSM ± SE | 0.46 ± 0.79 | 0.00 ± 0.79 | 0.69 |
| LSM difference [95% CI] | -0.46 [-2.84, 1.92] | |  |
| NPI-Q: Total distress score, LSM ± SE | -0.09 ± 0.68 | -0.52 ± 0.68 | 0.67 |
| LSM difference [95% CI] | -0.43 [-2.49, 1.64] | |  |
| Blood pressure |  |  |  |
| SBP (mmHg), LSM ± SE | -2.07 ± 5.02 | -4.70 ± 5.02 | 0.72 |
| LSM difference [95% CI] | -2.63 [-17.82, 12.56] | |  |
| DBP (mmHg), LSM ± SE | -1.18 ± 3.82 | -3.28 ± 3.82 | 0.71 |
| LSM difference [95% CI] | -2.11 [-13.67, 9.45] | |  |
| Serum lipid levels |  |  |  |
| TC (mg/dL), LSM ± SE | 0.07 ± 5.63 | -7.37 ± 5.63 | 0.38 |
| LSM difference [95% CI] | -7.44 [-24.49, 9.61] | |  |
| HDL-C (mg/dL), LSM ± SE | 0.77 ± 1.79 | 0.38 ± 1.79 | 0.89 |
| LSM difference [95% CI] | -0.38 [-5.82, 5.05] | |  |
| LDL-C (mg/dL), LSM ± SE | -4.62 ± 4.82 | -12.38 ± 4.82 | 0.28 |
| LSM difference [95% CI] | -7.75 [-22.34, 6.84] | |  |
| TG (mg/dL), LSM ± SE | -10.50 ± 10.55 | 7.27 ± 10.55 | 0.26 |
| LSM difference [95% CI] | 17.78 [-14.17, 49.73] | |  |
| MDA-LDL (U/L), LSM ± SE | 21.98 ± 7.38 | -2.52 ± 7.38 | 0.03 |
| LSM difference [95% CI] | -24.50 [-46.84, -2.16] | |  |
| Blood glucose levels |  |  |  |
| FPG (mg/dL), LSM ± SE | 1.66 ± 10.57 | 1.49 ± 10.57 | 0.99 |
| LSM difference [95% CI] | -0.17 [-32.19, 31.85] | |  |
| HbA1c (%), LSM ± SE | 0.16 ± 0.08 | 0.04 ± 0.08 | 0.27 |
| LSM difference [95% CI] | -0.13 [-0.36, 0.10] | |  |

ANCOVA, analysis of covariance; CI, confidence interval; DBP, diastolic blood pressure; FPG, fasting plasma glucose; HbA1c, hemoglobin A1c;HDL-C, high-density lipoprotein cholesterol; LDL-C, low-density lipoprotein cholesterol; LSM, least square mean; MDM-LDL, malondialdehyde-modified low-density lipoprotein ; NPI-Q, Neuropsychiatric Inventory Questionnaire; PPS, per protocol set; SBP, systolic blood pressure; SE, standard error; TC, total cholesterol; TG, triglycerides
